# 1-{3-[(7-Fluoro-9*H*-pyrimido[4,5-*b*]indol-4-yl)(meth­yl)amino]­piperidin-1-yl}propan-1-one

**DOI:** 10.1107/S2414314621001590

**Published:** 2021-02-23

**Authors:** Stanislav Andreev, Dieter Schollmeyer, Pierre Koch

**Affiliations:** a Institute of Pharmaceutical Sciences, Department of Pharmaceutical and Medicinal Chemistry, Eberhard Karls Universität Tübingen, Auf der Morgenstelle 8, 72076 Tübingen, Germany; b Department of Organic Chemistry, Johannes Gutenberg Universität Mainz, Duesbergweg 10-14, 55099 Mainz, Germany; Goethe-Universität Frankfurt, Germany

**Keywords:** crystal structure, pyrimido­indole, kinase inhibitor

## Abstract

The title compound, C_19_H_22_FN_5_O, has been synthesized as an inhibitor of glycogen synthase kinase-3β. Two mol­ecules inter­act *via* two N—H⋯N hydrogen bonds, forming centrosymmetric dimers.

## Abstract 2

The title compound has been synthesized as an inhibitor of glycogen synthase kinase-3β. Two mol­ecules inter­act *via* two N—H⋯N hydrogen bonds.

## Graphical abstract

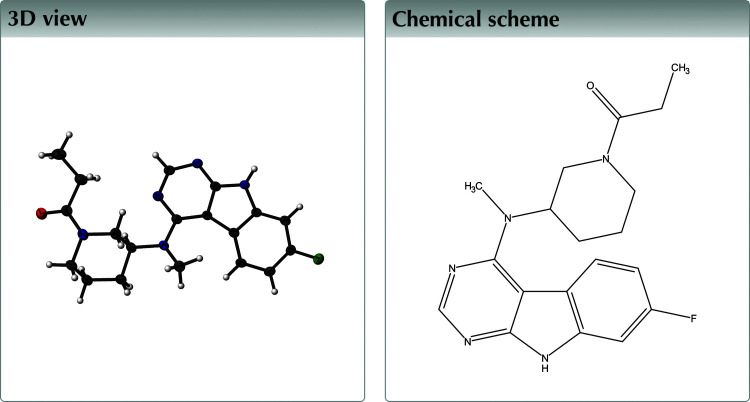

## Structure description

**Figure 1:**
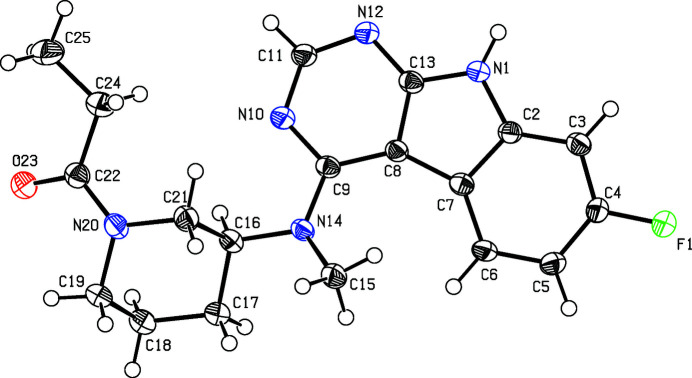
Perspective view of the title compound. Displacement ellipsoids are drawn at the 50% probability level.

**Figure 2:**
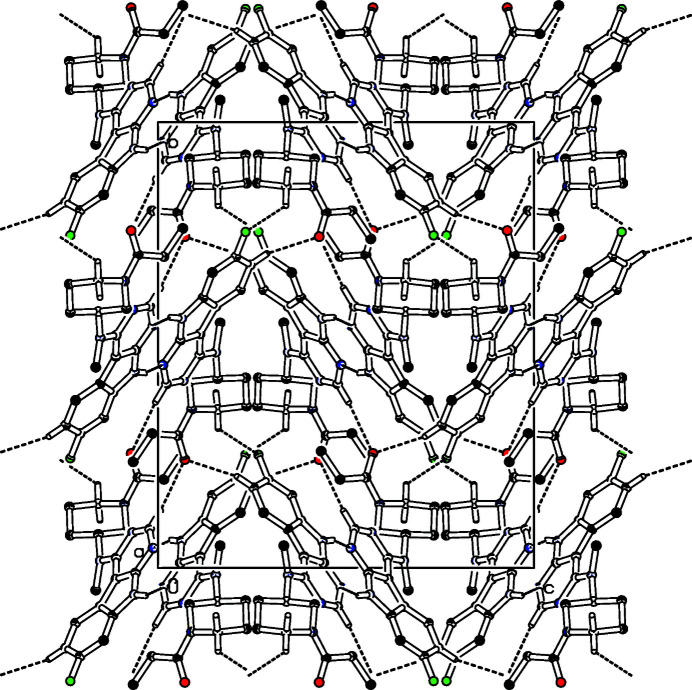
Partial packing diagram of the title compound, view along the *a* axis. Hydrogen bonds are indicated with dashed lines. Only hydrogen atoms involved in hydrogen bonds are shown.

The title compound, Fig. 1 ▸, was synthesized by amide coupling of 7-fluoro-*N*-methyl-*N*-(piperidin-3-yl)-9*H*-pyrimido[4,5-*b*]indol-4-amine and propionic acid and inhibits the glycogen synthase kinase-3β in the low micromolar range (Andreev *et al.*, 2020 ▸). The central 13-membered ring is nearly planar with a maximum deviation of 0.137 (2) Å while the piperidine ring has a chair conformation. Two mol­ecules form centrosymmetric dimers connected by two N—H⋯N hydrogen bonds (Table 1 ▸) while a three-dimensional network is formed *via* C—H⋯O and C—H⋯F hydrogen bonds (Table 1 ▸, Fig. 2 ▸).

## Synthesis and crystallization

The title compound was synthesized according to Andreev *et al.* (2020 ▸). Crystals for the X-ray analysis precipitated from a solution of the title compound in DMSO-*d*_6_ at 298 K.

## Refinement

Crystal data, data collection and structure refinement details are summarized in Table 2 ▸.

## Supplementary Material

Crystal structure: contains datablock(s) I, global. DOI: 10.1107/S2414314621001590/bt4108sup1.cif

Structure factors: contains datablock(s) I. DOI: 10.1107/S2414314621001590/bt4108Isup2.hkl

Supporting information file. DOI: 10.1107/S2414314621001590/bt4108Isup3.cml

CCDC reference: 2062292

Additional supporting information:  crystallographic information; 3D view; checkCIF report

## Figures and Tables

**Table 1:** Hydrogen-bond geometry (Å, °)

*D*—H⋯*A*	*D*—H	H⋯*A*	*D*⋯*A*	*D*—H⋯*A*
N1—H1⋯N12^i^	0.88	1.99	2.849 (3)	164
C5—H5⋯O23^ii^	0.95	2.47	3.380 (3)	161
C11—H11⋯O23^iii^	0.95	2.54	3.482 (3)	174
C19—H19*A*⋯O23	0.99	2.37	2.764 (3)	103
C19—H19*A*⋯F1^iv^	0.99	2.49	3.246 (3)	133

Symmetry codes: (i) 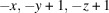 ; (ii) 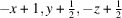 ; (iii) 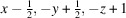 ; (iv) 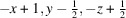 .

**Table 2:** Experimental details

Crystal data	
Chemical formula	C_19_H_22_FN_5_O
*M*_r_	355.41
Crystal system, space group	Orthorhombic, *P* *b* *c* *a*
Temperature (K)	120
*a*, *b*, *c* (Å)	10.6982 (3), 19.8473 (8), 16.7642 (6)
*V* (Å^3^)	3559.5 (2)
*Z*	8
Radiation type	Mo *K*α
μ (mm^−1^)	0.09
Crystal size (mm)	0.35 × 0.17 × 0.08

Data collection	
Diffractometer	Stoe IPDS 2T
No. of measured, independent and observed [*I* > 2σ(*I*)] reflections	9882, 4202, 2840
*R*_int_	0.066
(sin θ/λ)_max_ (Å^−1^)	0.658

Refinement	
*R*[*F* ^2^ > 2σ(*F* ^2^)], *wR*(*F* ^2^), *S*	0.069, 0.194, 1.14
No. of reflections	4202
No. of parameters	237
H-atom treatment	H-atom parameters constrained
Δρ_max_, Δρ_min_ (e Å^−3^)	0.36, −0.41

Computer programs: *X-AREA* *WinXpose*, *Recipe* and *X-AREA* *Integrate* (Stoe & Cie, 2019 ▸), *SHELXT2014* (Sheldrick, 2015*a* ▸) and *SHELXL2018/3* (Sheldrick, 2015*b* ▸).

## full crystallographic data

### Crystal data

**Table:**

C_19_H_22_FN_5_O	*D*_x_ = 1.326 Mg m^−^^3^
*M**_r_* = 355.41	Mo *K*α radiation, λ = 0.71073 Å
Orthorhombic, *P**b**c**a*	Cell parameters from 7975 reflections
*a* = 10.6982 (3) Å	θ = 2.4–28.3°
*b* = 19.8473 (8) Å	µ = 0.09 mm^−^^1^
*c* = 16.7642 (6) Å	*T* = 120 K
*V* = 3559.5 (2) Å^3^	Block, colourless
*Z* = 8	0.35 × 0.17 × 0.08 mm
*F*(000) = 1504	

### Data collection

**Table:**

Stoe IPDS 2T diffractometer	2840 reflections with *I* > 2σ(*I*)
Radiation source: sealed X-ray tube, 12 x 0.4 mm long-fine focus	*R*_int_ = 0.066
Detector resolution: 6.67 pixels mm^-1^	θ_max_ = 27.9°, θ_min_ = 2.4°
rotation method, ω scans	*h* = −14→12
9882 measured reflections	*k* = −22→26
4202 independent reflections	*l* = −19→22

### Refinement

**Table:**

Refinement on *F*^2^	Primary atom site location: dual
Least-squares matrix: full	Hydrogen site location: inferred from neighbouring sites
*R*[*F*^2^ > 2σ(*F*^2^)] = 0.069	H-atom parameters constrained
*wR*(*F*^2^) = 0.194	*w* = 1/[σ^2^(*F*_o_^2^) + (0.0822*P*)^2^ + 2.3561*P*] where *P* = (*F*_o_^2^ + 2*F*_c_^2^)/3
*S* = 1.14	(Δ/σ)_max_ = 0.001
4202 reflections	Δρ_max_ = 0.36 e Å^−^^3^
237 parameters	Δρ_min_ = −0.41 e Å^−^^3^
0 restraints	

### Special details

**Table:**

Geometry. All esds (except the esd in the dihedral angle between two l.s. planes) are estimated using the full covariance matrix. The cell esds are taken into account individually in the estimation of esds in distances, angles and torsion angles; correlations between esds in cell parameters are only used when they are defined by crystal symmetry. An approximate (isotropic) treatment of cell esds is used for estimating esds involving l.s. planes.
Refinement. H atoms found in a difference map. Then, they were positioned geometrically and refined using a riding model with C—H = 0.95 Å and *U*_iso_(H) = 1.2*U*eq(C) for aromatic CH, C—H = 0.99 Å and *U*_iso_(H) = 1.2*U*eq(C) for CH_2_, C—H = 1.00 Å and *U*_iso_(H) = 1.2*U*eq(C) for tertiary H and with C—H = 0.98 Å and *U*_iso_(H) = 1.5*U*eq(C) for CH_3_. The methyl groups were allowed to rotate but not to tip.

### Fractional atomic coordinates and isotropic or equivalent isotropic displacement parameters (Å^2^)

**Table:**

	*x*	*y*	*z*	*U*_iso_*/*U*_eq_	
F1	0.03835 (15)	0.75389 (8)	0.26711 (10)	0.0344 (4)	
N1	0.0797 (2)	0.56013 (11)	0.43722 (12)	0.0247 (5)	
H1	0.011506	0.562102	0.466503	0.030*	
C2	0.1147 (2)	0.60634 (12)	0.38014 (14)	0.0224 (5)	
C3	0.0481 (2)	0.66282 (13)	0.35527 (14)	0.0243 (5)	
H3	−0.028161	0.676063	0.379652	0.029*	
C4	0.1002 (2)	0.69815 (13)	0.29309 (15)	0.0260 (5)	
C5	0.2099 (3)	0.67981 (13)	0.25432 (16)	0.0272 (5)	
H5	0.239394	0.705203	0.210137	0.033*	
C6	0.2757 (2)	0.62393 (13)	0.28097 (15)	0.0259 (5)	
H6	0.351084	0.610925	0.255311	0.031*	
C7	0.2302 (2)	0.58663 (12)	0.34613 (14)	0.0221 (5)	
C8	0.2673 (2)	0.52527 (12)	0.38809 (14)	0.0213 (5)	
C9	0.3654 (2)	0.47773 (12)	0.38893 (14)	0.0218 (5)	
N10	0.3530 (2)	0.42059 (11)	0.43277 (13)	0.0255 (5)	
C11	0.2515 (2)	0.41287 (13)	0.47767 (15)	0.0256 (5)	
H11	0.245845	0.371894	0.506735	0.031*	
N12	0.15664 (19)	0.45588 (11)	0.48670 (12)	0.0250 (5)	
C13	0.1679 (2)	0.51100 (13)	0.44078 (14)	0.0230 (5)	
N14	0.47433 (18)	0.48402 (10)	0.34654 (12)	0.0225 (4)	
C15	0.5310 (2)	0.54981 (13)	0.33467 (16)	0.0283 (6)	
H15A	0.612776	0.551050	0.360984	0.042*	
H15B	0.476882	0.584631	0.357578	0.042*	
H15C	0.541595	0.558115	0.277441	0.042*	
C16	0.5572 (2)	0.42482 (13)	0.34154 (15)	0.0252 (5)	
H16	0.503095	0.383843	0.338129	0.030*	
C17	0.6387 (3)	0.42663 (14)	0.26687 (16)	0.0289 (6)	
H17A	0.691916	0.467432	0.267526	0.035*	
H17B	0.585136	0.428388	0.218752	0.035*	
C18	0.7214 (3)	0.36317 (14)	0.26433 (16)	0.0308 (6)	
H18A	0.668171	0.322908	0.256490	0.037*	
H18B	0.779753	0.366247	0.218634	0.037*	
C19	0.7956 (2)	0.35565 (14)	0.34126 (15)	0.0291 (6)	
H19A	0.842958	0.312818	0.340020	0.035*	
H19B	0.856282	0.393090	0.345791	0.035*	
N20	0.7121 (2)	0.35610 (11)	0.41082 (13)	0.0275 (5)	
C21	0.6384 (2)	0.41798 (13)	0.41636 (15)	0.0272 (6)	
H21A	0.694825	0.457341	0.420976	0.033*	
H21B	0.584720	0.416496	0.464414	0.033*	
C22	0.6811 (2)	0.29723 (13)	0.44725 (15)	0.0261 (5)	
O23	0.72866 (19)	0.24321 (10)	0.42863 (11)	0.0328 (5)	
C24	0.5879 (3)	0.30135 (14)	0.51579 (16)	0.0317 (6)	
H24A	0.610597	0.339869	0.550410	0.038*	
H24B	0.503685	0.310256	0.493727	0.038*	
C25	0.5829 (3)	0.23828 (16)	0.56585 (18)	0.0421 (8)	
H25A	0.554350	0.200474	0.532942	0.063*	
H25B	0.524624	0.244955	0.610271	0.063*	
H25C	0.666367	0.228412	0.586876	0.063*	

### Atomic displacement parameters (Å^2^)

**Table:**

	*U*^11^	*U*^22^	*U*^33^	*U*^12^	*U*^13^	*U*^23^
F1	0.0314 (8)	0.0340 (9)	0.0379 (9)	0.0069 (7)	0.0044 (7)	0.0117 (7)
N1	0.0215 (10)	0.0274 (11)	0.0252 (10)	0.0027 (9)	0.0058 (8)	0.0051 (8)
C2	0.0205 (11)	0.0266 (13)	0.0202 (11)	0.0000 (10)	0.0010 (9)	0.0013 (9)
C3	0.0201 (11)	0.0290 (13)	0.0238 (12)	−0.0001 (10)	0.0015 (10)	−0.0001 (10)
C4	0.0242 (12)	0.0254 (13)	0.0285 (12)	0.0026 (10)	−0.0004 (10)	0.0052 (10)
C5	0.0257 (13)	0.0279 (13)	0.0279 (13)	−0.0023 (11)	0.0026 (10)	0.0062 (10)
C6	0.0198 (12)	0.0311 (14)	0.0267 (12)	−0.0010 (10)	0.0048 (10)	0.0042 (10)
C7	0.0200 (11)	0.0243 (12)	0.0220 (11)	−0.0020 (9)	0.0009 (9)	−0.0014 (9)
C8	0.0177 (11)	0.0255 (12)	0.0207 (11)	0.0003 (9)	0.0004 (9)	0.0011 (9)
C9	0.0181 (11)	0.0262 (12)	0.0210 (11)	−0.0019 (10)	0.0000 (9)	−0.0005 (9)
N10	0.0202 (10)	0.0286 (11)	0.0276 (11)	0.0000 (9)	0.0027 (8)	0.0040 (8)
C11	0.0209 (11)	0.0300 (14)	0.0260 (12)	−0.0015 (10)	0.0013 (10)	0.0048 (10)
N12	0.0215 (10)	0.0268 (11)	0.0268 (11)	−0.0005 (9)	0.0029 (8)	0.0049 (8)
C13	0.0176 (11)	0.0293 (14)	0.0220 (12)	−0.0016 (10)	−0.0005 (9)	0.0004 (9)
N14	0.0174 (10)	0.0234 (11)	0.0266 (10)	−0.0002 (8)	0.0038 (8)	0.0002 (8)
C15	0.0212 (12)	0.0285 (14)	0.0352 (14)	−0.0009 (10)	0.0026 (11)	0.0026 (11)
C16	0.0204 (12)	0.0285 (13)	0.0266 (12)	0.0023 (10)	0.0032 (10)	0.0004 (10)
C17	0.0276 (13)	0.0321 (14)	0.0269 (13)	0.0036 (11)	0.0057 (11)	0.0041 (10)
C18	0.0299 (14)	0.0337 (15)	0.0288 (13)	0.0047 (12)	0.0072 (11)	−0.0003 (11)
C19	0.0244 (12)	0.0332 (14)	0.0299 (13)	0.0020 (11)	0.0075 (10)	0.0008 (11)
N20	0.0242 (11)	0.0306 (12)	0.0277 (11)	0.0049 (9)	0.0029 (9)	−0.0007 (9)
C21	0.0241 (12)	0.0295 (14)	0.0279 (13)	0.0018 (10)	−0.0003 (10)	−0.0004 (10)
C22	0.0239 (12)	0.0298 (14)	0.0246 (12)	0.0007 (10)	−0.0011 (10)	−0.0006 (10)
O23	0.0362 (11)	0.0302 (10)	0.0320 (10)	0.0063 (9)	0.0065 (8)	0.0003 (8)
C24	0.0318 (14)	0.0364 (15)	0.0270 (13)	0.0003 (12)	0.0057 (11)	−0.0014 (11)
C25	0.0496 (19)	0.0461 (19)	0.0305 (15)	0.0016 (15)	0.0114 (13)	0.0051 (13)

### Geometric parameters (Å, º)

**Table:**

F1—C4	1.361 (3)	C15—H15C	0.9800
N1—C13	1.359 (3)	C16—C17	1.526 (3)
N1—C2	1.377 (3)	C16—C21	1.532 (4)
N1—H1	0.8800	C16—H16	1.0000
C2—C3	1.392 (3)	C17—C18	1.540 (4)
C2—C7	1.417 (3)	C17—H17A	0.9900
C3—C4	1.375 (3)	C17—H17B	0.9900
C3—H3	0.9500	C18—C19	1.522 (4)
C4—C5	1.389 (4)	C18—H18A	0.9900
C5—C6	1.388 (4)	C18—H18B	0.9900
C5—H5	0.9500	C19—N20	1.469 (3)
C6—C7	1.406 (3)	C19—H19A	0.9900
C6—H6	0.9500	C19—H19B	0.9900
C7—C8	1.461 (3)	N20—C22	1.360 (3)
C8—C13	1.411 (3)	N20—C21	1.462 (3)
C8—C9	1.411 (3)	C21—H21A	0.9900
C9—N10	1.358 (3)	C21—H21B	0.9900
C9—N14	1.370 (3)	C22—O23	1.227 (3)
N10—C11	1.330 (3)	C22—C24	1.523 (4)
C11—N12	1.335 (3)	C24—C25	1.508 (4)
C11—H11	0.9500	C24—H24A	0.9900
N12—C13	1.343 (3)	C24—H24B	0.9900
N14—C15	1.453 (3)	C25—H25A	0.9800
N14—C16	1.474 (3)	C25—H25B	0.9800
C15—H15A	0.9800	C25—H25C	0.9800
C15—H15B	0.9800		
			
C13—N1—C2	108.6 (2)	C17—C16—C21	110.5 (2)
C13—N1—H1	125.7	N14—C16—H16	107.6
C2—N1—H1	125.7	C17—C16—H16	107.6
N1—C2—C3	127.3 (2)	C21—C16—H16	107.6
N1—C2—C7	109.4 (2)	C16—C17—C18	109.4 (2)
C3—C2—C7	123.2 (2)	C16—C17—H17A	109.8
C4—C3—C2	115.5 (2)	C18—C17—H17A	109.8
C4—C3—H3	122.2	C16—C17—H17B	109.8
C2—C3—H3	122.2	C18—C17—H17B	109.8
F1—C4—C3	117.4 (2)	H17A—C17—H17B	108.2
F1—C4—C5	118.3 (2)	C19—C18—C17	110.9 (2)
C3—C4—C5	124.3 (2)	C19—C18—H18A	109.5
C6—C5—C4	119.2 (2)	C17—C18—H18A	109.5
C6—C5—H5	120.4	C19—C18—H18B	109.5
C4—C5—H5	120.4	C17—C18—H18B	109.5
C5—C6—C7	119.7 (2)	H18A—C18—H18B	108.1
C5—C6—H6	120.1	N20—C19—C18	110.8 (2)
C7—C6—H6	120.1	N20—C19—H19A	109.5
C6—C7—C2	118.0 (2)	C18—C19—H19A	109.5
C6—C7—C8	136.0 (2)	N20—C19—H19B	109.5
C2—C7—C8	105.9 (2)	C18—C19—H19B	109.5
C13—C8—C9	114.8 (2)	H19A—C19—H19B	108.1
C13—C8—C7	105.3 (2)	C22—N20—C21	124.2 (2)
C9—C8—C7	139.8 (2)	C22—N20—C19	120.0 (2)
N10—C9—N14	116.1 (2)	C21—N20—C19	112.5 (2)
N10—C9—C8	119.4 (2)	N20—C21—C16	109.1 (2)
N14—C9—C8	124.5 (2)	N20—C21—H21A	109.9
C11—N10—C9	118.8 (2)	C16—C21—H21A	109.9
N10—C11—N12	127.7 (2)	N20—C21—H21B	109.9
N10—C11—H11	116.2	C16—C21—H21B	109.9
N12—C11—H11	116.2	H21A—C21—H21B	108.3
C11—N12—C13	112.8 (2)	O23—C22—N20	122.4 (2)
N12—C13—N1	123.2 (2)	O23—C22—C24	120.7 (2)
N12—C13—C8	126.2 (2)	N20—C22—C24	116.9 (2)
N1—C13—C8	110.6 (2)	C25—C24—C22	113.5 (2)
C9—N14—C15	120.5 (2)	C25—C24—H24A	108.9
C9—N14—C16	117.9 (2)	C22—C24—H24A	108.9
C15—N14—C16	117.21 (19)	C25—C24—H24B	108.9
N14—C15—H15A	109.5	C22—C24—H24B	108.9
N14—C15—H15B	109.5	H24A—C24—H24B	107.7
H15A—C15—H15B	109.5	C24—C25—H25A	109.5
N14—C15—H15C	109.5	C24—C25—H25B	109.5
H15A—C15—H15C	109.5	H25A—C25—H25B	109.5
H15B—C15—H15C	109.5	C24—C25—H25C	109.5
N14—C16—C17	111.8 (2)	H25A—C25—H25C	109.5
N14—C16—C21	111.4 (2)	H25B—C25—H25C	109.5
			
C13—N1—C2—C3	177.1 (2)	C2—N1—C13—C8	2.7 (3)
C13—N1—C2—C7	−0.5 (3)	C9—C8—C13—N12	−3.8 (4)
N1—C2—C3—C4	−175.6 (2)	C7—C8—C13—N12	174.0 (2)
C7—C2—C3—C4	1.7 (4)	C9—C8—C13—N1	178.6 (2)
C2—C3—C4—F1	−179.8 (2)	C7—C8—C13—N1	−3.6 (3)
C2—C3—C4—C5	1.7 (4)	N10—C9—N14—C15	−146.1 (2)
F1—C4—C5—C6	178.7 (2)	C8—C9—N14—C15	35.4 (3)
C3—C4—C5—C6	−2.7 (4)	N10—C9—N14—C16	9.8 (3)
C4—C5—C6—C7	0.4 (4)	C8—C9—N14—C16	−168.7 (2)
C5—C6—C7—C2	2.7 (4)	C9—N14—C16—C17	154.3 (2)
C5—C6—C7—C8	176.5 (3)	C15—N14—C16—C17	−49.0 (3)
N1—C2—C7—C6	173.9 (2)	C9—N14—C16—C21	−81.5 (3)
C3—C2—C7—C6	−3.9 (4)	C15—N14—C16—C21	75.2 (3)
N1—C2—C7—C8	−1.7 (3)	N14—C16—C17—C18	−179.1 (2)
C3—C2—C7—C8	−179.4 (2)	C21—C16—C17—C18	56.2 (3)
C6—C7—C8—C13	−171.2 (3)	C16—C17—C18—C19	−54.0 (3)
C2—C7—C8—C13	3.1 (3)	C17—C18—C19—N20	54.8 (3)
C6—C7—C8—C9	5.8 (5)	C18—C19—N20—C22	101.4 (3)
C2—C7—C8—C9	−179.9 (3)	C18—C19—N20—C21	−58.9 (3)
C13—C8—C9—N10	6.0 (3)	C22—N20—C21—C16	−98.9 (3)
C7—C8—C9—N10	−170.8 (3)	C19—N20—C21—C16	60.4 (3)
C13—C8—C9—N14	−175.5 (2)	N14—C16—C21—N20	176.0 (2)
C7—C8—C9—N14	7.7 (5)	C17—C16—C21—N20	−59.1 (3)
N14—C9—N10—C11	177.4 (2)	C21—N20—C22—O23	164.3 (2)
C8—C9—N10—C11	−4.0 (4)	C19—N20—C22—O23	6.4 (4)
C9—N10—C11—N12	−1.0 (4)	C21—N20—C22—C24	−18.4 (4)
N10—C11—N12—C13	3.2 (4)	C19—N20—C22—C24	−176.4 (2)
C11—N12—C13—N1	176.8 (2)	O23—C22—C24—C25	11.8 (4)
C11—N12—C13—C8	−0.6 (4)	N20—C22—C24—C25	−165.5 (3)
C2—N1—C13—N12	−175.1 (2)		

### Hydrogen-bond geometry (Å, º)

**Table:**

*D*—H···*A*	*D*—H	H···*A*	*D*···*A*	*D*—H···*A*
N1—H1···N12^i^	0.88	1.99	2.849 (3)	164
C5—H5···O23^ii^	0.95	2.47	3.380 (3)	161
C11—H11···O23^iii^	0.95	2.54	3.482 (3)	174
C19—H19*A*···O23	0.99	2.37	2.764 (3)	103
C19—H19*A*···F1^iv^	0.99	2.49	3.246 (3)	133

Symmetry codes: (i) −*x*, −*y*+1, −*z*+1; (ii) −*x*+1, *y*+1/2, −*z*+1/2; (iii) *x*−1/2, −*y*+1/2, −*z*+1; (iv) −*x*+1, *y*−1/2, −*z*+1/2.
